# Activation of ABA Receptors Gene *GhPYL9-11A* Is Positively Correlated with Cotton Drought Tolerance in Transgenic *Arabidopsis*

**DOI:** 10.3389/fpls.2017.01453

**Published:** 2017-08-23

**Authors:** Chengzhen Liang, Yan Liu, Yanyan Li, Zhigang Meng, Rong Yan, Tao Zhu, Yuan Wang, Shujing Kang, Muhammad Ali Abid, Waqas Malik, Guoqing Sun, Sandui Guo, Rui Zhang

**Affiliations:** ^1^Biotechnology Research Institute, Chinese Academy of Agricultural Sciences Beijing, China; ^2^College of Agronomy and Biotechnology, Southwest University Chongqing, China; ^3^Genomics Lab, Department of Plant Breeding and Genetics, Bahauddin Zakariya University Multan, Pakistan

**Keywords:** ABA, ABA receptor, GhPYL9, PP2C, seed germination, drought stress, cotton

## Abstract

The sensitivity to abscisic acid (ABA) by its receptors, pyrabactin resistance-like proteins (PYLs), is considered a most important factor in activating the ABA signal pathway in response to abiotic stress. However, it is still unknown which PYL is the crucial ABA receptor mediating response to drought stress in cotton (*Gossypium hirsutum* L.). Here, we reported the identification and characterization of highly induced ABA receptor GhPYL9-11A in response to drought in cotton. It is observed that *GhPYL9-11A* was highly induced by ABA treatment. GhPYL9-11A binds to protein phosphatase 2Cs (PP2Cs) in an ABA-independent manner. Moreover, the GhPYL-11A-PP2C interactions are partially disrupted by mutations, proline (P84) and histidine (H111), in the gate-latch region. Transgenic *Arabidopsis* overexpressing *GhPYL9-11A* plants were hypersensitive to ABA during seed germination and early seedling stage. Further, the increased in root growth and up regulation of drought stress-related genes in transgenic *Arabidopsis* as compared to wild type confirmed the potential role of *GhPYL9-11A* in abiotic stress tolerance. Consistently, the expression level of *GhPYL9-11A* is on average higher in drought-tolerant cotton cultivars than in drought-sensitive cottons under drought treatment. In conclusion, the manipulation of *GhPYL9-11A* expression could be a useful strategy for developing drought-tolerant cotton cultivars.

## Introduction

Abscisic acid (ABA) is a multifunctional phytohormone that regulates multiple aspects of plant growth and development, including seed dormancy and germination (Fang et al., 2008), vegetative development (Finkelstein et al., 2002), root elongation (Xing et al., 2016), leaf senescence (Liang et al., 2014), and fruit ripening (Zhang et al., 2009). ABA has also long been known to be a key endogenous messenger in the response to abiotic and biotic stresses, such as drought, salinity, cold, and pathogen infection (Choi et al., 2000; Xiong et al., 2002; Zhu, 2002; Sheard and Zheng, 2009; Yoshida et al., 2014). ABA-regulated stress responses include changes in gene expression, increased stomatal closure, reduced transpiration rate, protection of photosynthesis, and regulation of plant growth.

Previous studies have demonstrated that pyrabactin resistance-like (PYL), clade A protein phosphatase 2C (PP2C), and sucrose non-fermenting 1- related protein kinase 2 (SnRK2) proteins are the three core components of the ABA signaling cascade (Park et al., 2009; Sheard and Zheng, 2009; Zhu, 2016). The primary step in initiating the ABA signaling pathway is triggered by the PYL ABA receptors (Sheard and Zheng, 2009), which contain a central hydrophobic ligand-binding pocket (Iyer et al., 2001). In the absence of ABA, two β-loops, termed the gate and latch, together with several nearby structural elements form a large open pocket for ligand binding. When ABA is present, ABA binds to this pocket, leading to a series of conformational rearrangements, including gate and latch closure, which sequester ABA within the pocket. The ABA-bound PYL receptor physically interacts with and sequesters the constitutive SnRK2 repressor, PP2C, allowing SnRK2 kinases autophosphorylation. ABA-activated SnRK2 kinases subsequently phosphorylate and activate downstream ABA-responsive element-binding transcription factors that induce the expression of ABA-responsive genes (Melcher et al., 2009; Miyazono et al., 2009; Nishimura et al., 2009).

The PYL proteins belong to the START/Bet V1 superfamily (Iyer et al., 2001). There are fourteen PYL members in *Arabidopsis*, and this family is divided into three subfamilies based on amino acid sequences similarity (Zhao et al., 2013, 2016). All PYL proteins have highly conserved amino-acid residues in key interaction domains, including ABA-binding pocket, and the two entrance loops. Despite these similar structural features, PYLs have distinct properties and different expression patterns that explain the versatility of ABA signaling in plant growth and development. For example, *Arabidopsis* PYR1, PYL1, and PYL2 are able to form dimers in solution, whereas PYL proteins 4–10 exist as monomers (Hao et al., 2011). PYR1, PYL1, PYL2, and PYL3 interact with several clade A PP2Cs in an ABA-dependent manner. In contrast, most other PYL proteins except for PYL7 inhibit PP2Cs independently of ABA (Bai et al., 2013; Li et al., 2013; Zhao et al., 2013). Recently, several genetic studies have elucidated the significance of these receptors in ABA response; *pyr1 pyl1 pyl2 pyl4* quadruple mutants show ABA insensitivity (Park et al., 2009; Nishimura et al., 2010; Gonzalez-Guzman et al., 2012), and a *pyl8* single mutant has altered ABA responses during seed germination and seedling growth, as well as decreased drought resistance (Antoni et al., 2013; Zhao et al., 2014). PYL9 enhances drought resistance by limiting transpiration water loss and regulating senescence in old leaves and growth in young tissues (Zhao et al., 2016). Moreover, both PYL9 and PYL8 act as critical regulators of lateral root formation in response to ABA (Xing et al., 2016).

Upland cotton (*Gossypium hirsutum* L.) is an important economic crop and is an important source of fiber and oil (Wang et al., 2012, 2017; Shan et al., 2014). Compared with the model plants *Arabidopsis* and rice, cotton has higher tolerance to drought and salt stress (Liang et al., 2016). However, its growth and development, as well as fiber yield and quality are also significantly affected by severe environmental conditions, especially drought stress (He et al., 2016). To date, a number of candidate genes for drought tolerance in cotton have been identified. Several stress-related genes, including *GhABF2* (Liang et al., 2016), *GhNAC2* (Gunapati et al., 2016), *GhATAF1* (He et al., 2016), *GhbHLH130* (Guang et al., 2014), *GhSARP1* (Liu et al., 2016), *GhMKK3* (Wang C. et al., 2016), *Di19-1* and *Di19-2* (Qin et al., 2016), have been shown to regulate stress response in an ABA-dependent manner. Therefore, understanding ABA signal transduction in cotton will accelerate the molecular breeding of stress-tolerant cotton cultivars.

Because ABA has an essential role in plant growth and development, understanding how each PYL protein affects plant physiology has both basic and applied agricultural significance. Although there have been a number of studies of ABA receptors in the model plant *Arabidopsis* and other crops, such as rice and soybean, functional analysis of PYLs in upland cotton has not been reported. Upland cotton is a typical allotetraploid crop that was formed about 1–2 MYA by hybridization between an A-genome ancestor and a D-genome ancestor (Chen et al., 2007; Lin et al., 2010; Paterson et al., 2012; Li et al., 2014; Cao, 2015; Yuan et al., 2015), and the *PYL* gene family expanded in this process. In this study, we carried out genome-wide analysis to identify all *PYL* genes in the two diploid progenitor species, *G. raimondii* and *G. arboretum*, and two tetraploids *G. hirsutum* and *G. barbadense*. Genome-wide expression profiling analysis revealed that *GhPYL9-11A* was the most highly expressed PYL gene during drought stress in upland cotton. Consistent with a function in drought stress response, overexpression of *GhPYL9-11A* in *Arabidopsis* remarkably increases ABA sensitivity and enhances drought tolerance. The future characterization of *GhPYL9-11A* will further our understanding of ABA-mediated stress response in cotton, and facilitate its application in breeding drought tolerant cotton cultivars.

## Materials and Methods

### Plant Materials and Growth Conditions

Upland cotton (*G. hirsutum*) seeds of cultivar Y18R, a restores variety developed by our lab (Shi et al., 2012), were planted during the regular cotton cultivation season in 2015 and 2016 in the experimental farm at the Biotechnology Research Institute, Chinese Academy of Agricultural Sciences (Beijing, China). The recommended cultural and plant measures were adopted during the growth of cotton crop. For expression analysis, various organs including leaf, root, stem, and seed, were collected after 1 week of the onset of flowering stage. For analysis of ABA-induced *GhPYL9-11A* expression, cotton plants were grown in the Greenhouse with a 12-h-light (30°C)/12-h-dark (26°C) photoperiod with about 300 μM m^-2^s^-1^ photon density and 45% humidity, and the leaves of 3-weeks old plants were sprayed with 100 μM ABA (Sigma–Aldrich, United States). Ten plants were collected at 0, 1, 3, and 6 h after treatment and stored at -70°C for RNA isolation.

*Arabidopsis thaliana* Col-0 seed and *GhPYL9-11A* transgenic plants were sterilized for approximately 10 min in 10% bleach and then rinsed in sterile deionized water three times. Sterilized seeds were sown on petri dishes containing 0.8% agar media with aaa12 MS nutrients and 1% sucrose. After stratification at 4°C for 2 days, the dishes were moved to a growth chamber at 22°C with a 16-h light/8-h dark cycle and 70% humidity. All seedlings were grown vertically before transplanting to media supplemented different concentrations of ABA and mannitol.

### Gene Cloning and Expression Analysis

The full-length open reading frame of *GhPYL9-11A* was amplified from *G. hirsutum* L. using the Invitrogen RACE system (Invitrogen, United States). Total RNA was extracted using the EASYspin reagent (YPHBio, China). After DNase treatment, approximately 2 μg total RNA was reverse-transcribed using the ReverTra Ace qPCR RT Master Kit (Toyobo, Japan). Quantitative RT-PCR (qRT-PCR) analysis was performed using SYBR Green I PCR mix (SSoFast EvaGreen Supermix, Bio-Rad, United States) and the Bio-Rad CFX96 real time PCR system. Data were analyzed with Opticon monitor software (Bio-Rad). The cotton *GhHISTON3* (*GhHIS3*) and *Arabidopsis Actin 8* genes, both with stability expression in the different tissues, developmental stages and environmental conditions (Zhu et al., 2017), were used as internal controls. Primers used are listed in **Table S1**. Values are means ± SD of three biological repeats. Student’s *t*-test was used for statistical analysis.

### Vector Construction and *Arabidopsis* Transformation

The *GhPYL9-11A* full-length coding sequence was cloned into *pBI121-35S* to generate the *pBI121-35S:GhPYL9-11A* overexpression construct. The constructs were transfected into *Agrobacterium tumefaciens* GV3101 by electroporation, and then transformed into *Arabidopsis* Col-0 pants using the floral dipping method (Liu L. et al., 2015). Homozygous transgenic *Arabidopsis* lines were obtained, and the lines GO16, GO21, and GO22, which have high levels of *GhPYL9-11A* expression, were selected for further analysis. The primers used for vector construction are listed in **Table S1**.

### Yeast Two-Hybrid Assay

The Y2H assay was performed using the Matchmaker^TM^ Gold Yeast Two-hybrid System (Clontech, United States). Full length cDNA of *GhPYL9-11A* was amplified and cloned into the pGBKT7 vector, and *AtABI1*, *AtABI2*, *GhPP2C1*, and *GhPP2C2* full length cDNAs were amplified and cloned into the pGADT7 vector. *GhPYL9-11A* point mutations were introduced by site-directed mutagenesis using *pBI121-35S:GhPYL9-11A* as a template, and *GhPYL9-11A^P84S^* and *GhPYL9-11A^H111A^* were cloned into the pGBKT7 vector. Each pair of AD and BD constructs was co-transformed according to the manufacturer’s protocol. The positive yeast liquid culture was serially diluted to OD600 = 0.6 and 3 μL of dilutions 1:10, 1:100, 1:1,000, and 1:10,000 were inoculated onto various plates [Synthetic Dropout Medium (SD) -leucine (Leu)/tryptophan (Trp), SD-Leu/Trp/histidine (His), and SD-Leu/Trp/His supplemented with 10 μM ABA]. All plates were incubated at 28°C for 2 days and then photographed.

### Germination Assay

Surface-sterilized seeds were sown on aaa12 MS without or with ABA (0.3 and 0.5 μM) or mannitol (150 and 300 μM). After 5 days seeds with green shoots were scored as germinated.

### Drought Stress Treatment

To test the drought tolerance of *35S:GhPYL9-11A* transgenic *Arabidopsis*, the homozygous F2 generation was subjected to drought stress in soil in a growth chamber. Seed from GO1, GO2, GO3, and wild type lines were imbibed at 4°C for 2 days and directly planted in soil in the same pots (30 cm in diameter). All plants were grown at room temperature (22–25°C) under long-day conditions (16 h light/8 h dark) in the culture room. After 2 weeks of seedling emergence, drought treatment was established by withholding the water for 4 weeks. The experiment was comprised of five replications. Watering was resumed 1 day after the 4 weeks of drought treatment, and the survival rate (SR) was calculated 3 days later.

### Chlorophyll Measurement

After measuring the fresh weight of the samples, five seedlings for each sample were used to calculated the total chlorophyll contents as the absorbance at 652 nm following (Liang et al., 2014).

### Photosynthesis Parameters and Water Loss Measurement

For measurement of photosynthesis parameters, water was withheld for 7 days beginning when plants were 14-days old. Ten independent plants were used for each *GhPYL9-11A* overexpression line. Photosynthesis parameters were measured as previously described by (Liang et al., 2015). For determination of water loss, the whole aboveground rosettes of 21-day-old plants were cut from the base and weighed at different time points.

### Electrolyte Leakage Measurement

The measurement of electrolyte leakage was performed according to a previously described method (Lin A. et al., 2012). Ten different leaf disks from wild-type and *GhPYL9-11A* overexpression plants were placed into a flask containing 10 mL distilled deionized water. After shaking at room temperature for 6 h at approximately 120 rpm, the conductivity (*C*_i_) was measured with a conductivity meter (Leici-DDS-307A). Then, the disks were boiled for 20 min to kill the leaf tissues and shaken for 1 h to completely release the electrolytes into the solution. The conductivity (*C*_m_) was measured again. The electrolyte leakage was calculated according to the equation (*C*_i_/*C*_m_) × 100%.

### Hydrogen Peroxide Production and Measurement of Antioxidant Enzyme Activities

For hydrogen peroxide production and measurement of antioxidant enzyme activities, *Arabidopsis* plants were grown on soil for 14 days and leaves were collected from the plants after water was withheld for 7 days. Measurement of H_2_O_2_ production was performed according to a previously described method (Liang et al., 2015), and quantified using the Hydrogen peroxidase assay kit (Beyotime, China) according to the manufacturer’s instructions. The quantification of CAT, SOD, and POD activity was performed according to a previously described method (Liang et al., 2016). Total protein was measured using Bradford protein assay kit (Sangon Biotech, China).

### Protein Sequence and Phylogenetic Analysis

For phylogenetic analysis of GhPYL9-11A homologs in plants, 20 protein sequences were obtained through National Center for Biotechnology Information database using BLASTp searches with the GhPYL9-11A protein as a query. All sequences were aligned using Clustal W. Phylogenetic trees were constructed using MEGA 6.0 based on the neighbor-joining method. Topological robustness was assessed by bootstrap analysis with 1,000 replicates (Liu L. et al., 2015).

## Results

### Transcriptome Analysis of Cotton PYL ABA Receptor

**FIGURE 1:**
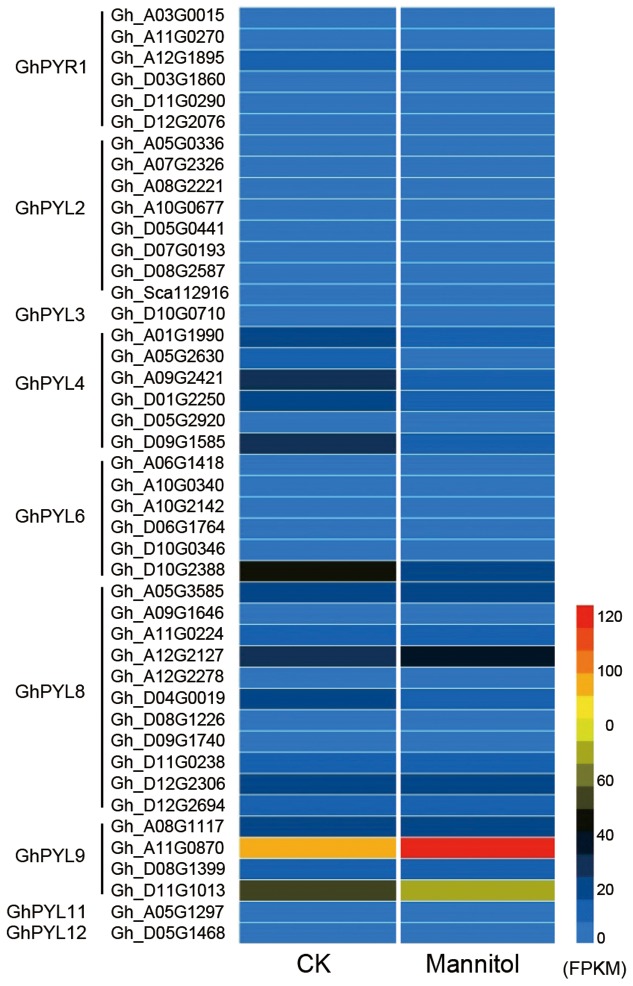
Heat map of pyrabactin resistance-like (PYL) family genes in upland cotton. Detailed information about gene annotations can be found in **Table S1**. Gene annotation data were obtained from the Cotton Functional Genomics Database (https://cottonfgd.org/).

Pyrabactin resistance-like protein coding sequences were identified in upland cotton species, *G. hirsute*, *G. barbadense, G. raimondii*, and *G. arboretum*, with complete genomes or genome assemblies. We then compared the expression profiles of the *PYL* genes under drought stress. Twenty-two PYL proteins were identified from each of the two diploid progenitor species *G. raimondii* and *G. arboretum*, and 44 and 36 members were identified in the tetraploids *G. hirsutum* and *G. barbadense*, respectively (**Table S2**). High-resolution temporal expression profiling revealed that two *GhPYL* genes, *Gh_A11G08070* and *Gh_D11G1013*, were most highly expressed during drought stress in upland cotton (*G. hirsutum*) (**Figure 1** and **Table S2**). The proteins encoded by *Gh_A11G0870* and *Gh_D11G1013* share approximately 80 and 79% amino acid identity with AtPYL9, an ABA receptor, respectively.

Both Gh_A11G0870 and Gh_D11G1013 belong to the PYL9 subfamily of PYL proteins. There are two members each in *G. raimondii* and *G. arboretum*, and four members each in *G. hirsutum* and *G. barbadense*. Based on phylogenetic analysis, the PYL proteins could be further divided into two distinct subgroups, PYL9-I and PLY9-II (**Figure S1**). Interestingly, all members of PYL9-I in both diploid and tetraploid cotton were highly expressed during seed germination and drought stress, whereas PLY9-II members were with low expression level (**Table S2**). These results suggest that PYL9-I proteins may play important roles in the regulation of seed germination and drought responses in cotton. Because Gh_A11G08070 (hereafter referred to as GhPYL9-11A) belongs to the drought stress-responsive PYL9-I subfamily, its function was investigated in detail.

### Structural Features of GhPYL9-11A

**FIGURE 2:**
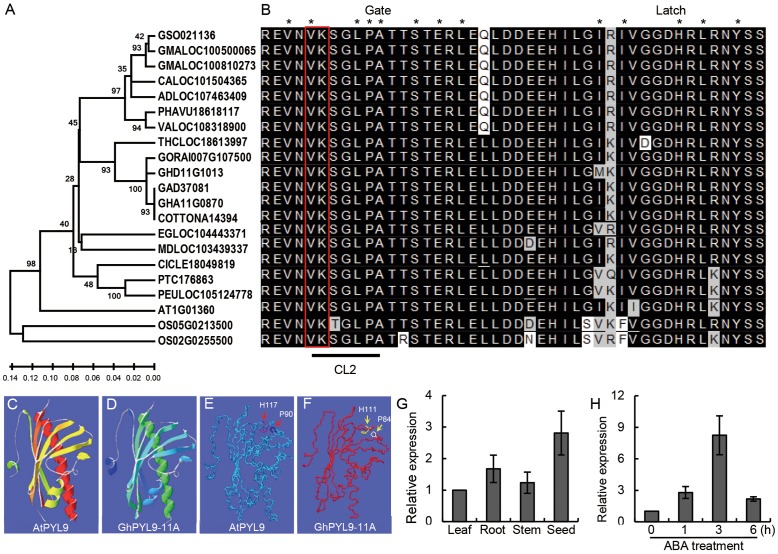
Phylogenetic analyses of *GhPYL9-11A* homologs and organic-specific expression of *GhPYL9-11A*. **(A)** Phylogenetic relationship of *GhPYL9-11A* homologs in plants. AT (*Arabidopsis thaliana*), OS (*Oryza sativa*), GSO (*Glycine soja*), GMA (*Glycine max*), CAL (*Citrus clementina*), AD (*Arachis duranensis*), PHAVU (*Phaseolus vulgaris*), VA (*Vigna angularis*), THC (*Theobroma cacao*), GORA (*Gossypium raimondii*), GH (*Gossypium hirsutum*), GA (*Gossypium arboreum*), EG (*Eucalyptus grandis*), MD (*Malus domestica*), CICLE (*Citrus clementine*), PT (*Populus trichocarpa*), PEU (*Populus euphratica*). **(B)** Amino acid alignment of the PYL9 CL2/gate-latch region for the proteins shown in **(A)**. The black line indicates the CL2 domain. Two amino acid residues ‘VK’ are marked with a red open rectangle. The residues in contact with the ABA molecule are designated by asterisk. **(C–F)** Comparison of the predicted 3-D structures of AtPYL9 and *GhPYL9-11A*. The conserved proline and histidine residues in the gate and latch region are marked with red arrows in AtPYL9 **(E)** and yellow arrows in *GhPYL9-11A* **(F)**. **(G)** Expression of *GhPYL9-11A* in various organs determined by quantitative real time (qRT)-PCR analysis. Roots, leaves, and stems were harvested from 3-week-old plants. **(H)** Time-course of the response of *GhPYL-11A* to 100 μM ABA treatment.

Using protein database searches, we identified a number of putative GhPYL9-11A orthologs in various higher plants. GhPYL9-11A homologs were only found in the plant kingdom, indicating that PYL9 proteins are plant-specific. The identification of PYL proteins in both monocots and dicots suggests that PYL proteins are evolutionally conserved across plant species. The existence of PYL9 in different plant species might also suggest that its function in drought stress response is also conserved. Phylogenetic analysis of the cotton GhPYL9-11A protein and its 20 homologs showed that GyhPYL9-11A is most closely related to LOC18613997 of *Theobroma cacao*, and shares approximately 80% amino acid sequence identity with related proteins in **Figure 2A**.

PYL9 family proteins have a pyrabactin resistance1/pyr1-like/regulatory component of ABA receptor (PYR/PYL/RCAC)-like structure (amino acids 28–172) typical of ABA receptors (**Figure 2B**). We analyzed the 3D structure of this conserved motif in GhPYL9-11A using SWISSMODEL. The predicted structure is a common helix-grip structural feature consisting of four α-helixes and seven antiparallel β-sheets and is highly similar to the *Arabidopsis* AtPYL9 PYR/PYL/RCAC motif (**Figures 2C,D**).

### Expression Pattern of *GhPYL9-11A*

Quantitative RT-PCR analysis showed that *GhPYL9-11A* is expressed preferentially in seeds and roots, with a low level of expression in the leaf and stem (**Figure 2E**). Moreover, time-course analysis revealed that *GhPYL9-11A* was rapidly induced by ABA, with expression levels increasing approximately 2.8-fold after 1 h of ABA application (**Figure 2F**). Levels of *GhPYL9-11A* transcription reached a maximum after 4 h of treatment, and were approximately eightfold higher than without hormone treatment. These results clearly demonstrate that this gene is transcribed in response to ABA and thus is potentially involved in ABA response.

### GhPYL9-11A Interacts with Clade A PP2Cs in an ABA-Independent Manner

**FIGURE 3:**
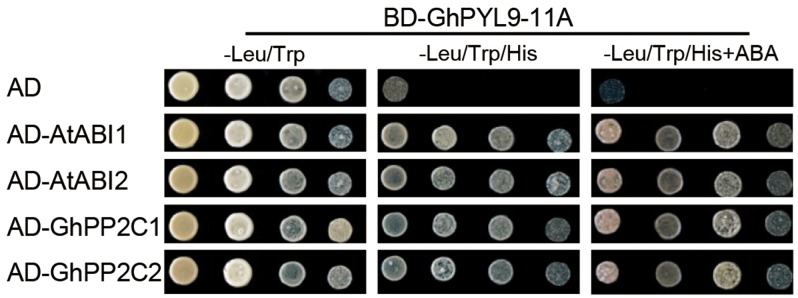
Interactions between *GhPYL9-11A* and PP2C proteins in yeast. The BD-GhPYL9-11A fusion was co-expressed with AD-AtABI1, AD-AtABI2, AD-GhPP2C1, or AD-GhPP2C2 in yeast. Interactions were determined by growth assays on media lacking leucine (Leu), tryptophan (Trp), and histidine (His) in the presence and absence of 10 μM ABA.

It has been shown that PYL proteins can interact with PP2Cs in yeast two-hybrid (Y2H) assays in an ABA-dependent or ABA-independent manner (Bai et al., 2013; Zhao et al., 2013). To investigate whether GhPYL9-11A binds clade A PP2Cs, we tested the interaction of GhPYL9-11A with two *Arabidopsis* PP2C proteins, *A. thaliana* ABSCISIC ACID INSENSITIVE1 (AtABI1) and AtABI2, in Y2H assays in the presence or absence of ABA. As shown in **Figure 3**, GhPYL9-11A interacted with both AtABI1 and AtABI2 in an ABA-independent manner. We further tested the interaction between GhPYL9-11A and two cotton PP2Cproteins, GhPP2C1 (Gh_A05G0308) and GhPP2C2 (Gh_A07G0123), that share high similarity with AtABI1 (63% for GhPP2C1 and 71% for GhPP2C2, **Figure S2**). GhPYL9-11A also binds to both GhPP2C1 and GhPP2C2 in an ABA-independent manner (**Figure 3**).

**FIGURE 4:**
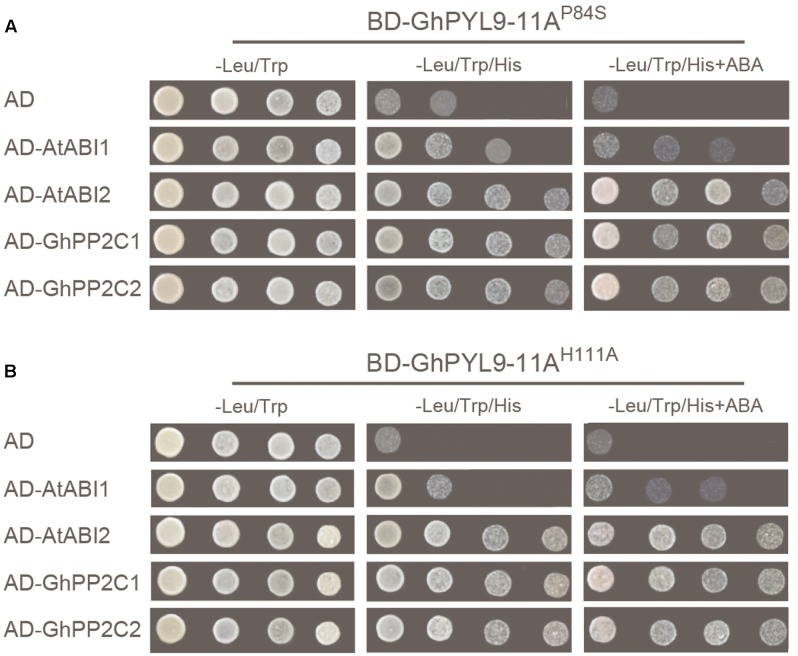
GhPYL-11A-PP2C interactions are partially mediated by proline (P84) and histidine (H111) in the gate-latch region of *GhPYL9-11A*. Replacement of P84 by serine (GhPYL9-11A^P84S^) **(A)** and H111 by alanine (GhPYL9-11A^H111A^) **(B)** abolished binding to AtABI1 both in the absence and presence of 10 μM ABA. However, these mutations partially affected binding to AtABI2, GhPP2C1, and GhPP2C2 both in the presence and absence of 10 μM ABA.

The mutated conserved proline (P88S) in the gate region and histidine (H111A) in the latch region of GhPYL9-11A, and tested the ability of the mutated proteins, GhPYL9-11A^P84S^ and GhPYL9-11A^H111A^, to interact with AtABI1, AtABI2, GhPP2C1, and GhPP2C2 in Y2H assays. Both the GhPYL9-11A^P84S^ and GhPYL9-11A^H111A^ mutations reduced the level of interaction with AtABI1, AtABI2, GhPP2C1, and GhPP2C2 both in the presence and absence of ABA (**Figure 4**). These data suggest that GhPYL-11A-PP2C interactions are partially mediated by P84 and H111 in the gate-latch region of GhPYL9-11A.

### Overexpression of *GhPYL9-11A* in *Arabidopsis* Leads to an ABA-Hypersensitive Phenotype during Germination

**FIGURE 5:**
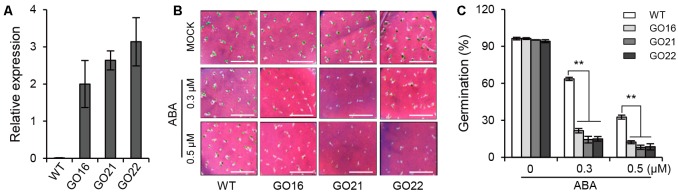
The effect of *GhPYL9-11A* overexpression on *Arabidopsis* seed germination. **(A)** The expression levels of *GhPYL9-11A* in transgenic *Arabidopsis* plants determined by qRT-PCR. GO, *GhPYL9-11A* overexpression lines. Values are means ± SD of three biological repeats. **(B)** Germinated seeds from wild-type plants and GO lines grown for 5 days on half-strength Murashige and Skoog (aaa12 MS) in the presence or absence of ABA. **(C)** Seed germination rate under the different growth conditions in **(B)**. Values are means ± SD of five measurements. ^∗∗^*P* ≤ 0.01; Student’s *t*-test.

To investigate the biological function of *GhPYL9-11A* in plants, the *GhPYL9-11A* coding sequence was cloned into the plant binary vector pBI121 in front of the 35S promoter and transformed into *Arabidopsis* though *Agrobacterium tumefaciens*-mediated transformation. A set of 27 positive independent transgenic lines had up-regulated expression of *GhPYL9-11A*. Among them, three independent homozygous T3 *GhPYL9-11A*-overexpressing (GO) lines, GO16, GO21, and GO22, with high levels of *GhPYL9-11A* transcript, were selected for further analysis (**Figure 5A**).

To determine whether GhPYL9-11A is a functional ABA receptor, we treated three GO plants and wild type with 0.3 and 0.5 μM ABA and then compared their seed germination phenotypes. Under control conditions, there was no significant difference in seed germination rate between GO plants and wild type. However, the seed germination rates of the three GO transgenic *Arabidopsis* lines were less than 20 and 10% in the presence of 0.3 μM ABA and 0.5 μ M ABA, respectively, whereas the germination rates of wild type were 63.5 and 32.6%, respectively (**Figures 5B,C**). These observations suggest that overexpression of *GhPYL9-11A* represses seed germination in the presence of ABA in *Arabidopsis*.

### *GhPYL9-11A* Overexpression in *Arabidopsis* Results in ABA-Hypersensitive Phenotypes during Seedling Growth

**FIGURE 6:**
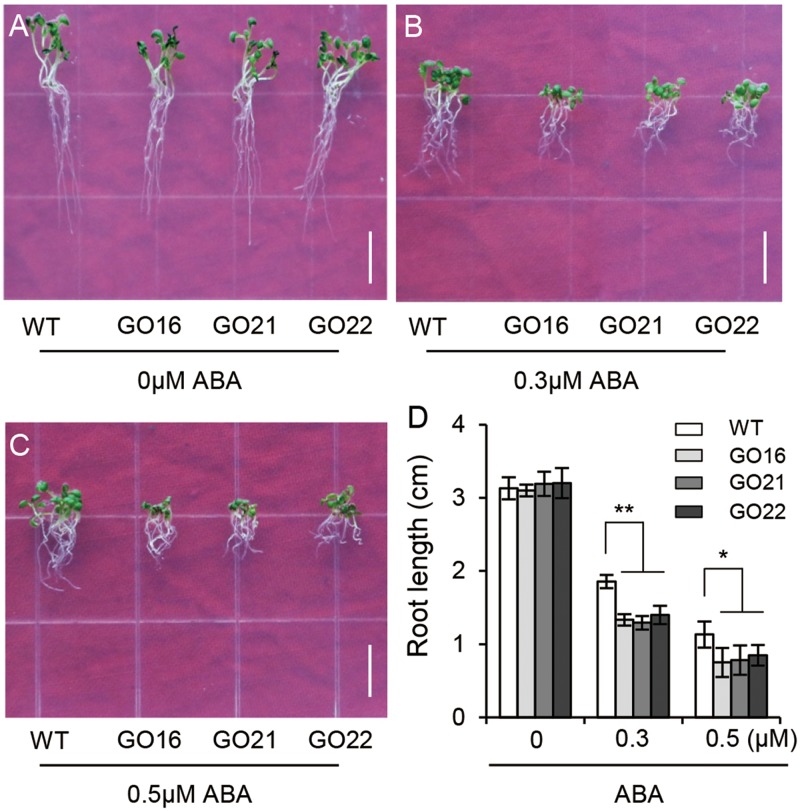
Differential ABA responsiveness of *GhPYL9-11A* overexpression and wild-type *Arabidopsis* seedlings. **(A–C)** Phenotypes of wild type and three independent *35S:GhPYL9-11A* transgenic *Arabidopsis* lines, GO16, GO21, and GO22, grown in aaa12 MS medium supplemented with 0 μM **(A)**, 0.3 μM **(B)**, and 0.5 μM **(C)** ABA. Bar = 1 cm. **(D)** Seedling primary roots lengths under the different growth conditions in **(A–C)**. Measurements were performed for least 30 independent seedlings. Results are shown as mean ± SD of three replicates; Student’s *t*-test. ^∗^*P* ≤ 0.05, ^∗∗^*P* ≤ 0.01.

We further examined the effects of *GhPYL9-11A*-overexpression on *Arabidopsis* seedling root growth. We found no obvious difference in root development between GO transgenic plants and wild type at different stages of seedling growth. However, ABA treatment repressed root growth to a larger extent in the GO lines compared with wild type; in the presence of 0.3 M ABA the root lengths of in the three GO lines were reduced by 37.5–47.9% compared with control conditions, but in wild type root lengths were only reduced by 28.8%. In the absence of ABA, and in the presence of 0.5 μM ABA, root lengths were 44.8–52.6% of GO plants while 34.8% of wild type (**Figures 6A–D**). These results suggest that overexpression of *GhPLY9-11A* in *Arabidopsis* significantly increases sensitivity to ABA. Thus, we deduced that GhPYL9-11A may act as an ABA receptor in cotton.

### The Seeds of *Arabidopsis* GO Lines Are Hypersensitive to Osmotic Stress

**FIGURE 7:**
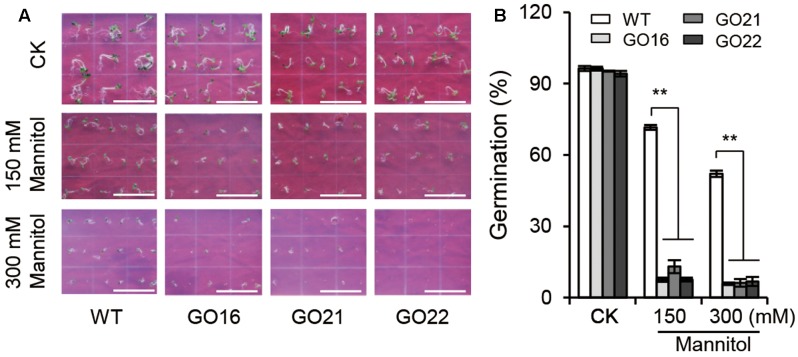
Effect of *GhPYL9-11A* overexpression on *Arabidopsis* seed germination under mannitol treatment. **(A)** Germinated seeds from wild-type and *GhPYL9-11A* overexpression plants grown for 5 days on 1/2 MS with or without mannitol. **(B)** Seed germination rate under the different growth conditions in **(A)**. Values are means ± SD of five measurements. ^∗∗^*P* ≤ 0.01; Student’s *t*-test.

Keeping in view the ability of ABA to regulate seed germination, we examined the effects of *GhPYL9-11A* overexpression on seed germination under osmotic stress imposed by adding 150 and 300 mM mannitol to aaa12 MS media. Compared with wild-type *Arabidopsis*, GO plants had a significantly decreased ratio of seed germination under osmotic stress (**Figure 7**), but no difference in seed germination rate between GO plants and wild type was observed under control conditions. The repression of seed germination under osmotic stress in the *GhPYL9-11A* overexpression lines is consistent with hypersensitivity to ABA.

### *GhPYL9-11A* Overexpression in *Arabidopsis* Enhances Tolerance to Drought Stress

**FIGURE 8:**
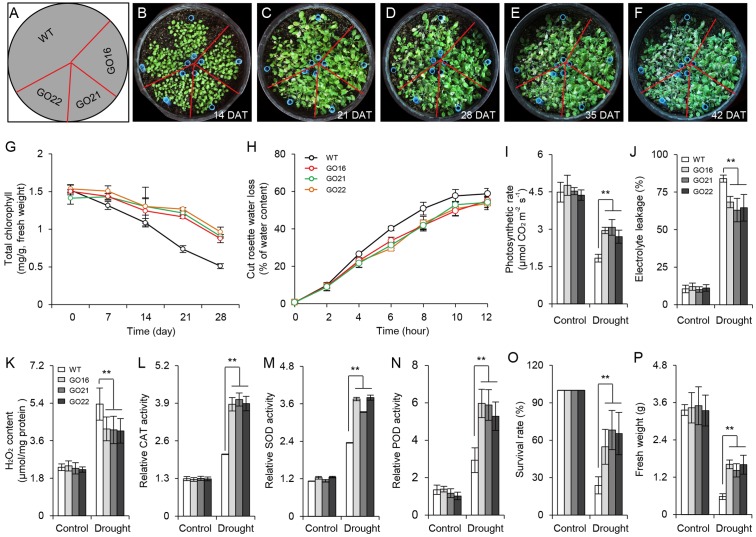
Transgenic *Arabidopsis* harboring *35S:GhPYL9-11A* exhibit increased drought tolerance. **(A–F)** *35S:GhPYL9-11A* confers drought tolerance to *Arabidopsis*. After 2 weeks of growth, water was withheld for 28 days under short-day conditions before watering was resumed. Representative images of plants taken 7, 14, 21, and 28 days after watering was withheld. DAT, day after transplanting. **(G)** Chlorophyll content of mature leaves in wild type and GO plants at the indicated times after watering were withheld. Values are means ± SD of 20 measurements; Student’s *t*-test. **(H)** Cumulative transpirational water loss from the rosettes of wild-type and GO *Arabidopsis* at the indicated times after detachment. Values are means ± SD of 20 measurements; Student’s *t*-test. **(I–N)** Physiological parameters of GO plants under drought stress treatment. Parameters were measured after water was withheld for 7 days. Overexpression of *GhPYL9-11A* in *Arabidopsis* increased the photosynthetic rate **(I)**, reduced electrolyte leakage **(J)**, reduced H_2_O_2_ content **(K)**, and increased the activities of antioxidant enzymes, including CAT **(L)**, SOD **(M)**, and POD **(N)**. Error bars indicate SD (*n* = 5). ^∗∗^*P* ≤ 0.01; Student’s *t*-test. **(O)** Survival rate (SR) of the wild-type and GO *Arabidopsis*. After 2 weeks of growth, plants were subjected to drought stress by withholding water for 4 weeks. Survival was scored 3 days after watering was resumed. Error bars indicate SD (*n* = 3); Student’s *t*-test. **(P)** Relative fresh weights of wild type and GO lines. After watering was withheld for 28 days, the rosettes were collected and weighted. Plants grown under well-watered conditions served as the control. Error bars indicate SD (*n* = 3). ^∗∗^*P* ≤ 0.01; Student’s *t*-test.

**FIGURE 9:**
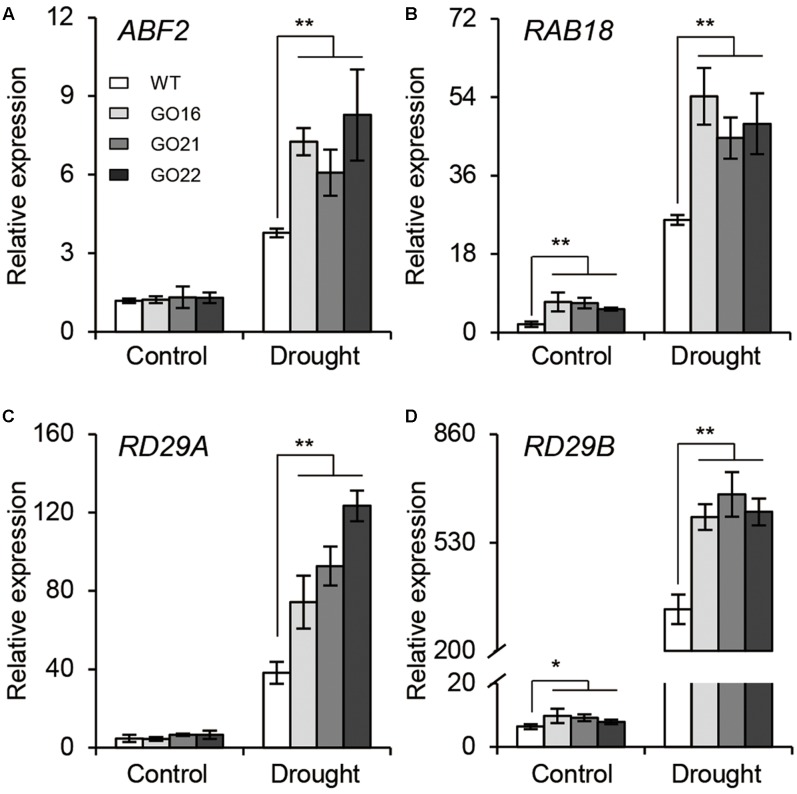
Expression of ABA-responsive genes in *GhPYL9-11A* overexpression plants under control and drought conditions. QRT-PCR analysis of ABA-responsive genes, including *ABF2* **(A)**, *RAB18* **(B)**, *DR29A* **(C)**, and *RD29B* **(D)**, in wild type and *Arabidopsis GhPYL9-11A* overexpression lines treated with 400 μM mannitol for 24 h. Values are means ± SD of three biological repeats. ^∗^*P* ≤ 0.05, ^∗∗^*P* ≤ 0.01; Student’s *t*-test.

The ABA-dependent signaling pathway has an essential role in response to drought stress therefore, we examined whether overexpression of *GhPYL9-11A* was sufficient to confer drought stress tolerance to *Arabidopsis*. The *Arabidopsis 35S:GhPYL9-11A* plants, GO16, GO21, and GO22, exhibited significantly increased drought tolerance during the vegetative stage (**Figures 8A–F**). GO plants also had reduced chlorophyll degradation (**Figure 8G**), reduced water loss (**Figure 8H**), enhanced photosynthetic rate (**Figure 8I**), reduced electrolyte leakage (**Figure 8J**), reduced accumulation of toxic hydrogen peroxide (**Figure 8K**), and enhanced activities of antioxidant enzymes including catalase (CAT, **Figure 8L**), superoxide dismutase (SOD, **Figure 8M**) and peroxidase (POD, **Figure 8N**). Consistent with these observations, the SR and total biomass were significantly increased after drought treatment in GO lines compared with the wild type (**Figures 8O,P**). Moreover, four ABA-dependent drought stress-associated genes, *ABF2*, *RAB18*, *RD29A*, and *RD29B*, were more strongly induced after drought treatment in the rosette leaves of *Arabidopsis* GO lines than in wild type (**Figures 9A–D**). Taken together, these data provide strong evidence that *GhPYL9-11A* overexpression confers drought tolerance in plants by activating the ABA-dependent signaling pathway.

### *GhPYL9-11A* Expression Level Associates with Drought Stress Tolerance in Different Cultivated Cotton Cultivars

**FIGURE 10:**
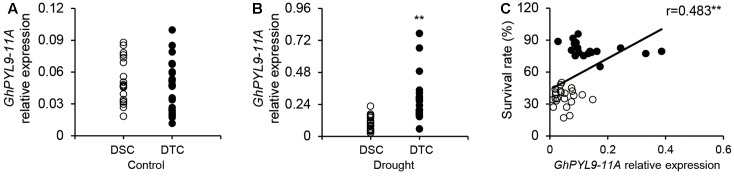
Expression level of *GhPYL-11A* in drought-tolerant and drought-sensitive cultivated cotton varieties is correlated with SR under severe drought stress. **(A,B)** The expression level of *GhPYL9-11A* in drought-tolerant and drought-sensitive cultivated cotton varieties treated with H_2_O **(A)** and 400 mM mannitol **(B)** for 24 h was determined by qRT-PCR. DSC, drought-sensitive cultivated cotton. DTC, drought-tolerant cultivated cotton. In **(A,B)**, asterisks indicate significant differences (^∗∗^*P* < 0.01). **(C)** Correlation between the SR of cotton seedlings grown under severe drought conditions and the expression level of *GhPYL9-11A*.

Our results clearly demonstrate that overexpression of *GhPYL9-11A* in *Arabidopsis* enhances drought stress tolerance. Thus, we speculated that the expression level of *GhPLY-11A* may be correlated with drought tolerance in cultivated cotton varieties. To test this hypothesis, we phenotyped the drought tolerance of 226 cotton varieties. These varieties were indexed by plant SR under severe drought stress, and 20 drought-tolerant (with SR ranging from 17.0 to 49.3%) and 20 drought-sensitive (with SR ranging from 65.2 to 95.9%) cotton varieties were selected for further analysis (**Table S3**). We next compared the seedling-stage expression levels of *GhPYL9-11A* in drought-tolerant and drought-sensitive cultivated cotton accessions under drought treatment. The average expression levels of *GhPYL9-11A* in the 20 drought-tolerant cotton varieties were significantly higher than in the drought-sensitive varieties under drought treatment (**Figure 10B**). However, the expression level did not differ under control conditions (**Figure 10A**). We further examined the relationship between the *GhPYL9-11A* expression levels and SR in 20 drought-tolerant and 20 drought-sensitive cultivated cotton varieties under drought stress and found that *GhPYL9-11A* expression is positively correlated with drought tolerance (*R* = 0.483; **Figure 10C**). These results further suggest that *GhPYL9-11A* plays an important role in drought tolerance in cultivated cotton.

## Discussion

Recently, significant progress has been made in the identification and characterization of the PYL ABA receptors in several plants (Sheard and Zheng, 2009; Bai et al., 2013). However, to our knowledge, up to now, no ABA receptors from upland cotton, which is widely grown in over 80 countries and provides the material for about 90% of the world’s cotton lint production (Wang et al., 2012; Wang Y. et al., 2016; Paterson and Wendel, 2015; Liang et al., 2016), have been reported. Owing to allopolyploidization, the number of *PYL* genes has doubled in upland cotton compared with diploid cotton. However, to date, it remains largely unknown which PYL members is the crucial ABA receptor mediating response to drought stress in tetraploid cotton. Therefore, it is interesting and important to determine the functions and regulatory mechanisms of the ABA receptors in tetraploid cotton. In this study, we identified 22 ABA receptors orthologous to *Arabidopsis* PYLs in both the A- and D-genomes of the tetraploid cotton *G. hirsutum* using the recently released cotton genome sequence (Wang et al., 2012; Li et al., 2014, 2015; Liu X. et al., 2015). We further demonstrated that GhPYL9-11A is a functional ABA receptor, and similar to AtPYL9, interacts with PP2Cs in ABA-independent manner and regulates drought resistance and leaf senescence in *Arabidopsis* (Zhao et al., 2016). We found that *GhPYL9-11A* overexpression in *Arabidopsis* results in ABA-hypersensitive seed germination and root development phenotypes. Moreover, overexpression of *GhPYL9-11A* also significantly enhances drought and osmotic stress tolerance.

Abscisic acid was discovered in the 1960s, and to date, numerous biochemical and genetic studies have revealed that ABA is a vital regulator of multiple stress responses (Hurst, 1995; Finkelstein et al., 2002; Zhu, 2002; Sheard and Zheng, 2009; Zhao et al., 2014). ABA enhances plant survival under stress conditions by inducing seed and bud dormancy, inhibiting germination, closing stomata, and accelerating old leaf senescence and abscission. A variety of abiotic stresses sharply increase endogenous ABA levels and activates ABA signaling pathways leading to elevated plant survival. Furthermore, exogenously applied ABA has been shown to induce the expression of genes known to enhance stress tolerance. Our previous studies have demonstrated that several ABA-responsive genes, such as *GhABF2* (Liang et al., 2016), *GhDr1* (Ding et al., 2011), and *GhSRK2D* (Lin F. et al., 2012), play important roles in ABA signal transduction in response to drought stress in cotton. In this study, through comparative analysis of the transcript levels of 44 *PYL* genes, we found that *GhPYL9-11A* (*Gh_A11G08070*) is the most highly expressed during both seed germination and drought stress (**Figure 1** and **Table S2**). Consistently, the homologs of *GhPYL9-11A*, *Gorai_007G107500* and *Cotton_A14394*, in diploid cotton *G. raimondii* and *G. arboreum*, respectively, also show highly expressed pattern during seed germination and drought stress (**Table S2**). Moreover, the expression level of *GhPYL9-11A* is associated with drought stress in different cultivated cotton cultivars (**Figure 10**). We also confirmed that expression of *GhPYL9-11A* is indeed significantly induced by exogenous ABA treatment (**Figure 2**). These findings suggest that among the cotton PYLs, *GhPYL9-11A* may be the most important regulator of ABA signaling during seed germination and under drought stress.

Pyrabactin resistance-likes are ABA receptors that act at the apex of a negative regulatory pathway and regulate ABA signaling by binding and inhibiting PP2Cs (Melcher et al., 2009; Miyazono et al., 2009; Nishimura et al., 2009; Park et al., 2009; Santiago et al., 2009; Sheard and Zheng, 2009). Similar to AtPYL-PP2C interactions in *Arabidopsis*, GhPYL9-11A interacts with AtABI1 and AtABI2, as well as with GhPP2C1 and GhPP2C2 in Y2H assays (**Figure 3**). Moreover, the interaction between GhPL9-11A and PP2C proteins does not depend on ABA. Previous studies have revealed that PYL-PP2C interactions are partially associated with two conserved amino acid residues preceding the CL2/gate-latch domain (**Figure 4**), and there are several polymorphisms in these residues, including VI, VV, VK, VQ, VT, VR, VN, IT, LV, and LK, in *Arabidopsis*, rice, soybean, poplar, and maize (Bai et al., 2013; Zhao et al., 2013). These polymorphisms are closely correlated with ABA-dependent or -independent interactions with PP2C. GhPYL9-11A belong to subgroup II of the GhPYL family, and have the typical VK combination in the CL2/gate-latch domain (**Figure 2B**). Consistent with the ABA-independent interaction between GhPYL0-11A and PP2Cs, VK is correlated with ABA-independent PYL-PP2C interactions. Intriguingly, *GhPYL9-11A* and AtPYL9 share high amino acid sequence identity and 3D structures, and both proteins contain the conserved proline (P84) and histidine (H111) residues in the gate-latch domain (**Figure 2C**). However, the site-directed mutants GhPYL9-11A^P84S^ and GhPYL9-11A^H111A^ are able to interact with AtABI2, GhPP2C1, and GhPP2C2, but not AtABI1 in an ABA-independent manner (**Figures 3**, **4**), suggesting that residues in other regions of GhPYL9-11A also regulate the ABA-independent interactions with PP2Cs in cotton.

To escape unfavorable environmental conditions at unusual times or of atypical severity, plants increase seed dormancy and inhibit growth by elevating ABA content and activating core ABA signaling pathways (Zhu, 2002; Zhao et al., 2016). Although recent studies have greatly improved our knowledge about the PYL family in several plants, the basic biological functions of PYLs in tetraploid cotton remain largely unknown. Here, we clearly demonstrate that *GhPYL9-11A* overexpression in *Arabidopsis* has multiple physiological effects, including enhanced ABA sensitivity and inhibition of seed germination under mannitol treatment (**Figures 3**, **4**). *GhPYL9-11A* overexpression also significantly increased seedling drought and osmotic tolerance, enhanced antioxidant enzyme activities, reduced the change in photosynthetic rate, reduced chlorophyll degradation, water loss, and electrolyte leakage, and induced the expression of a subset of ABA-regulated genes (**Figures 8**, **9**). The combination of inhibition of seed germination and enhanced seedling abiotic stress tolerance in *GhPYL9-11A* overexpression lines is consistent with plant survival. These results and the observation that drought tolerant cotton varieties have higher *GhPYL9-11A* expression levels suggests that *GhPYL9-11A* may have played a role in the evolution of drought and osmotic tolerance in cotton.

## Conclusion

GhPYL9-11A acts as a positive regulator of the ABA signaling pathway by binding to type PP2C protein phosphatases in an ABA-independent manner. Our study provides valuable insight into the function of GhPYL9-11A in the regulation of ABA signaling. In addition, the improved drought and osmotic tolerance of both seeds and seedlings of *Arabidopsis GhPYL9-11A* overexpression lines is encouraging for future efforts to genetically engineer plant drought and osmotic tolerance. Further manipulation of *GhPYL-11A* expression in cotton could aid the development of drought and osmotic tolerant cultivars.

## Author Contributions

CL, SG, and RZ designed research. CL, YLiu, YLi, ZM, RY, YW, SK, MA, and GS performed research. CL, TZ, and RZ analyzed data. CL, WM, SG, and RZ wrote the paper.

## Conflict of Interest Statement

The authors declare that the research was conducted in the absence of any commercial or financial relationships that could be construed as a potential conflict of interest.

## Supplementary Material

FIGURE S1 Phylogenetic analysis of PYL9 proteins in two diploid progenitor species *G. raimondii* and *G. arboretum*, and 4 two tetraploids *G. hirsutum* and *G. barbadense*. The scale bar indicates 0.1 amino acid substitution per site.

FIGURE S2 Protein sequence alignment of PP2C protein, AtABI1, AtABI2 GhPP2C1, and GhPP2C2 in *Arabidopsis* and cotton using Cluster X. Conserved amino acid are highlighted in shades of black and gray. White letter with black background (100% identity), white letter with gray background (60%).

TABLE S1 Primers used in this study.

TABLE S2 Expression pattern of PYL genes in cotton.

TABLE S3 Drought-tolerance and drought-sensitive cotton varieties used for correlation analysis between survival rate and *GhPYL9-11A* expression level.
