# Supplementary material for: Activation of ABA Receptors Gene GhPYL9-11A Is Positively Correlated with Cotton Drought Tolerance in Transgenic Arabidopsis
Source: Front Plant Sci. 2017 Aug 23;8:1453. doi: 10.3389/fpls.2017.01453 (PMC5572150; doi:10.3389/fpls.2017.01453)
Supplement: FIGURE S1 — Phylogenetic analysis of PYL9 proteins in two diploid progenitor species G. raimondii and G. arboretum, and 4 two tetraploids G. hirsutum and G. barbadense. The scale bar indicates 0.1 amino acid substitution per site. [file Presentation_1.pdf]

Supplementary Materials

Activation of ABA receptors gene *GhPYL9-11A* is positively correlated with cotton drought tolerance in transgenic *Arabidopsis*

Chengzhen Liang[1,†], Yan Liu[1,†], Yanyan Li[1,†], Zhigang Meng[1], Rong Yan[1,2], Tao Zhu[1], YuanWang[1], Shujing Kang[1], Muhammad Ali abid [1], Waqas Malik[1,3], Guoqing Sun[1], Sandui Guo[1,*] and Rui Zhang[1,*]

[1] Biotechnology Research Institute, Chinese Academy of Agricultural Sciences, Beijing, China

[2] College of Agronomy and Biotechnology, Southwest University, Chongqing, China

[3]Genomics Lab, Department of Plant Breeding and Genetics, Bahauddin Zakariya University, Multan, Pakistan

[†] Theses authors contributed equally to this article.

Running title: GhPYL9-11A enhances plant drought tolerance

[*]Correspondence:

Prof. Rui Zhang

E-mail: zhangrui@caas.cn

Prof. Sandui Guo

E-mail: guosandui@caas.cn

Supplementary Figure Legends

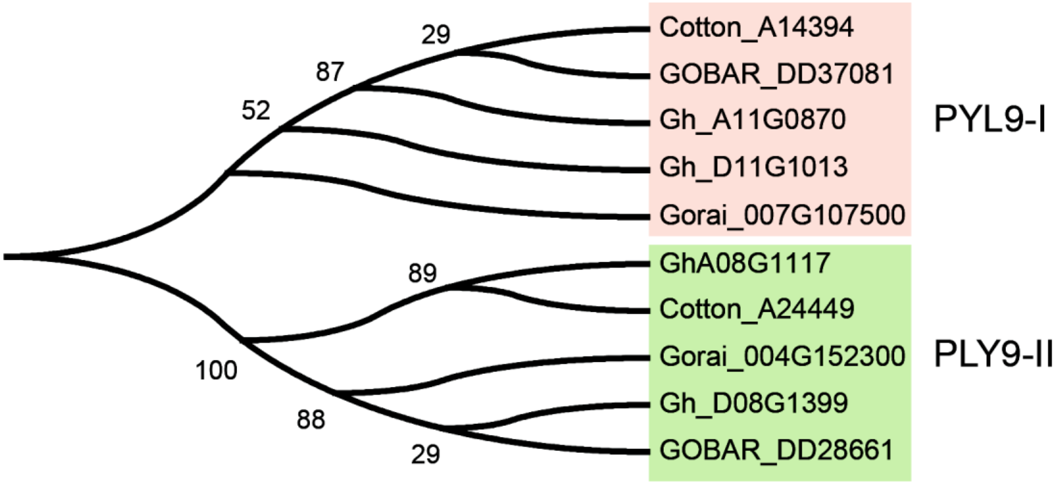

Figure S1. Phylogenetic analysis of PYL9 proteins in two diploid progenitor species *G.raimondii* and *G.arboretum*, and 4 two tetraploids *G. hirsutum* and *G. barbadense*. The scale bar indicates 0.1 amino acid substitution per site.

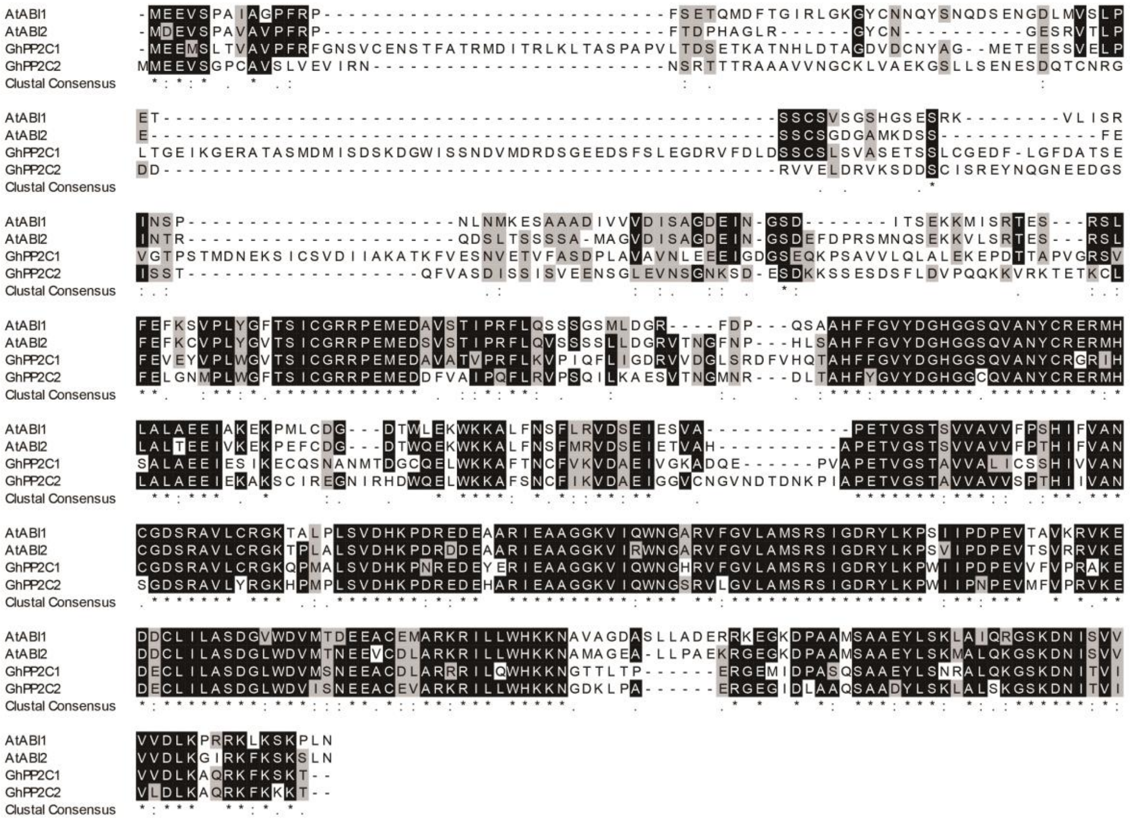

Figure S2. Protein sequence alignment of PP2C protein, AtABI1, AtABI2 GhPP2C1, and GhPP2C2 in *Arabidopsis* and cotton using Cluster X. Conserved amino acid are highlighted in shades of black and gray. White letter with black background (100% identity), white letter with gray background (60%).

Table S1. Primers used in this study.

	Forward Primers	Reverse Primers
**Primers Used for qRT-PCR**		
GhPYL9-11A	CAATCATAACGGTCCATCCA	AGAGCCTCCACAAAGTAACAAG
GhHIS	TCAAGACTGATTTGCGTTTCCA	GCGCAAAGGTTGGTGTCTTC
NPTII	GGAGTGAAAGAGCCTGATGC	CGGCTCCGTCGATACTATGT
RD29A	CAGGTGAATCAGGAGTTGTT	CCGGAAATTTATCCTCTTCT
RD29B	GCAAGCAGAAGAACCAATCA	CTTTGGATGCTCCCTTCTCA
ABF2	GTTTCAGCAGCCTTCTCCAC	CCACAAGACCACCACCTCTT
RAB18	ATCGGAGATTCTGGTGTTGG	TCTCTTGCCCAGCTGTATCC
AtActin8	AACTATGTTCTCAGGTATTG	TGGAAACGATGTCTCTTTAG
**Primers Used for Vector Construction**		
GhABF2ORFFP	GCGGCCGCATGCTTACGGTATGGTCATTGGT	CCTGCAGGGTACCCGTTGATAGGTTGTGTTTGG
GhPP2C1	CCCGGGATGGAGGAGATGTCTTTGACG	GAGCTCTCAGGTCTTACTCTTGAAC
GhPP2C2	CCCGGGATGATGGAAGAAGTATCTG	GAGCTCTCATGTTTTCTTCTTAAA
AtABI1	CCCGGGATGGAGGAAGTATCTCCGGC	GGATCCTCAGTTCAAGGGTTTGCTCTTG
AtABI2	CCCGGGATGGACGAAGTTTCTCCTGC	GAGCTCTCAATTCAAGGATTTGCTCTTG
GhPYL9P84S	GGTGGTGGCTGAAAGGCCAGATTTA	TCAGCCACCACCAGCACCGAAC
GhPYL9H111A	CGTCGCCTCCGACGATTTTCATG	GGAGGCGACGCTCGTCTCAGGAATT

Table S3. Drought-tolerance and drought-sensitive cotton varieties used for correlation analysis between survival rate and *GhPYL9-11A* expression level.

Accession name	Survival rate (%)
Huanghe A7	49.3
Huanghe B8	38.5
Huanghe A6	38.2
Changjiang A3	27.3
Xibei A3	34.6
Tianjin 9	35.4
Jiangsu A7	27.1
Xibei B5	41.9
Changjiang C3	35.2
Jiangsu A8	42.3
Huanghe E5	42.4
Jiangsu A1	45.7
Huanghe A9	41.7
Huanghe A8	48.3
Jiangsu A6	40.5
Huanghe C6	35.4
Huanghe A2	33.2
Zhengjiang 1207	17.2
Huanghe A6	19.3
Jaingsu A1	32.0

Accession name	Survival rate (%)
Huanghe E1	65.1
Xinhai 29	75.4
Changjiang C2	77.4
Huanghe C7	79.5
Xinhai 34	79.5
Henan 4	79.5
Jinagsu B8	78.4
Huanghe E4	77.4
Jiangsu A5	78.4
Changjiang C6	75.4
Henan 3	80.5
Huanghe A2	82.5
Tianjin 8	88.7
Henan 5	91.8
Changjiang A1	82.5
Nannong 10	95.9
Tianjin 6	87.7
Jiangsu B2	86.6
Xinluzhong 36	84.4
Jiangsu B1	90.2
